# Group sparse canonical correlation analysis for genomic data integration

**DOI:** 10.1186/1471-2105-14-245

**Published:** 2013-08-12

**Authors:** Dongdong Lin, Jigang Zhang, Jingyao Li, Vince D Calhoun, Hong-Wen Deng, Yu-Ping Wang

**Affiliations:** 1 Biomedical Engineering Department, Tulane University, New Orleans, LA, USA; 2 Center of Genomics and Bioinformatics, Tulane University, New Orleans, LA, USA; 3 Department of Biostatistics and Bioinformatics, Tulane University, New Orleans, LA, USA; 4 The Mind Research Network, Albuquerque, NM, 87131, USA; 5 Department of Electrical and Computer Engineering, University of New Mexico, Albuquerque, NM, 87131, USA

**Keywords:** Group sparse CCA, Genomic data integration, Feature selection, SNP

## Abstract

**Background:**

The emergence of high-throughput genomic datasets from different sources and platforms (*e.g.*, gene expression, single nucleotide polymorphisms (SNP), and copy number variation (CNV)) has greatly enhanced our understandings of the interplay of these genomic factors as well as their influences on the complex diseases. It is challenging to explore the relationship between these different types of genomic data sets. In this paper, we focus on a multivariate statistical method, canonical correlation analysis (CCA) method for this problem. Conventional CCA method does not work effectively if the number of data samples is significantly less than that of biomarkers, which is a typical case for genomic data (*e.g.*, SNPs). Sparse CCA (sCCA) methods were introduced to overcome such difficulty, mostly using penalizations with *l*-1 norm (CCA-*l*1) or the combination of *l*-1and *l*-2 norm (CCA-elastic net). However, they overlook the structural or group effect within genomic data in the analysis, which often exist and are important (*e.g.*, SNPs spanning a gene interact and work together as a group).

**Results:**

We propose a new group sparse CCA method (CCA-sparse group) along with an effective numerical algorithm to study the mutual relationship between two different types of genomic data (*i.e.*, SNP and gene expression). We then extend the model to a more general formulation that can include the existing sCCA models. We apply the model to feature/variable selection from two data sets and compare our group sparse CCA method with existing sCCA methods on both simulation and two real datasets (human gliomas data and NCI60 data). We use a graphical representation of the samples with a pair of canonical variates to demonstrate the discriminating characteristic of the selected features. Pathway analysis is further performed for biological interpretation of those features.

**Conclusions:**

The CCA-sparse group method incorporates group effects of features into the correlation analysis while performs individual feature selection simultaneously. It outperforms the two sCCA methods (CCA-*l*1 and CCA-group) by identifying the correlated features with more true positives while controlling total discordance at a lower level on the simulated data, even if the group effect does not exist or there are irrelevant features grouped with true correlated features. Compared with our proposed CCA-group sparse models, CCA-*l*1 tends to select less true correlated features while CCA-group inclines to select more redundant features.

## Background

In recent years, the development of a variety of affordable high throughput genome-wide assays enables multiple measurements of genomic markers from different platforms and/or scales for the same subject, *e.g.*, gene expression, single nucleotide polymorphisms (SNP), copy number variation, and proteomic data. Each of these measurements provides different but complementary information about genome variations. Combining multiple types of data not only can contribute to a better understanding of biological mechanism but also can have the potential to improve the diagnosis and treatments of complex diseases. Therefore, integrative approaches for large-scale genomic data analysis are in highly demand [1-3].

A number of approaches have been proposed to analyze multiple genomic data, *e.g.*, partial least squares correlation (PLSC [4]) and canonical correlation analysis (CCA [5]). CCA is closely related to PLSC [6], which is obtained by maximizing the correlation between the linear combinations of variables from two data sets, *e.g.*, a linear combination of SNPs and a linear combination of gene expressions. CCA can provide a global and thought view of data by compressing variables into 2 or 3 dimensions which are actually able to contain the dominant characteristics of data, for example, gene expressions involving in the same pathway process have the similar effect from the variations in multiple SNPs. It may hence provide better understanding of the underlying known or unknown biological systems. For example, the co-expressed and co-regulated genes and their associating SNPs. Different from the regression based integrative methods (*i.e.,* with principle component analysis and PLS), CCA focus on the canonical correlation framework without more prior knowledge of which type of omic data is explained or regressed by the another one (*e.g.,* with transcripts and metabolites). It is more applicable for exploring the correlated information from the paired omic data sets by CCA method. However, the conventional CCA model is not effective for the analysis of genomic data with small sample size because of the issue of high dimensionality-the number of biomarkers is always greatly larger than that of samples. An example is the SNP array, where millions of SNPs exist but only a few array samples are available. Conventional CCA will perform poorly in such a case [7-9]. In addition, this high dimensionality can result in possible multi-collinearity (linear dependence) problem, and thus computational difficulty [7].

To address the above issues, one way is to perform dimension reduction by using principle component analysis (PCA [10]) or independent component analysis (ICA [11]) followed by the conventional CCA. Another way is to introduce the sparsity penalty into the conventional CCA model, named sparse CCA (sCCA) by incorporating the feature selection into CCA to detect the correlation between a small subset of features. This is inspired by recent developments of sparse representation methods, *e.g.*, sparse linear regression (SLR [12]), sparse logistic regression [13], and sparse principle component analysis (SPCA [14]). To list a just few examples, Waaijenborg *et al.*[15] introduced the *l*-1 norm and elastic net penalties to the CCA model to analyze the correlation between gene expression and DNA-markers. Parkhomenko *et al.*[7] proposed a sCCA method with lasso penalty based on SVD (Singular value decomposition). Le Cao *et al.*[2] used the penalized CCA with the elastic net to identify sets of co-expressed genes from two different microarray platforms. Witten *et al.*[16] developed a penalized matrix decomposition (PMD) method and applied it to solve CCA with lasso and fuse lasso penalties. Generally, all of these sCCA models have the ability of identifying subsets of features by using sparse penalties such as the *l*-1 norm(denoted by *CCA*-*l1*) or elastic net (denoted by *CCA*-*elastic net*) [17,18]; however, they have not yet accounted for group structures within the data in the analysis, which often exist in genomic data. For example, genes within the same pathway have similar functions and act together in regulating a biological system. These genes can add up to have a larger effect and therefore can be detected as a group (*i.e.*, at a pathway or gene set level [19]).

Considering a group of features instead of individual feature has been found to be effective for biomarker identification [20-23]. Yuan and Lin proposed the group lasso for group variables selection [20]. Meier *et al.*[21] extended it to the logistic regression. Puig *et al.*[22] and Simon *et al.*[24] modified the group lasso to solve the non-orthonormal matrices. Although group lasso penalty can increase the power for variable selection, it requires a strong group-sparsity [25], and cannot yield sparsity within a group [26]. Friedman *et al.*[26] proposed a sparse group lasso penalty by combining the *l*-1 norm with group lasso to yield sparsity at both the group and individual feature level. Zhou *et al.*[27] applied it to genomic feature identification. Recently some work has been reported to incorporate the ‘group effect’ into a conventional CCA model. Chen *et*.*al* studied structure based CCA and proposed tree-based and network-based CCA [28]. Chen *etal.* incorporated the group effect into an association study of nutrient intake with human gut microbiome [29]. Both papers show an improvement when incorporating the group effect, however, a prior knowledge of group structure is needed and only the group effect of one type of data is discussed. Motivated by this, in this paper we develop a more general group sparse CCA method. This model has the following advantages: 1) feature selection will be performed at both group and single feature levels. Both irrelevant groups of features and individual features in the remaining groups will be removed at the same time, without prior knowledge of group structures; 2) it provides a general framework for canonical correlation analysis. It can be reduced to the CCA group model which can account for the group effect of multiple features. It can also revert to the popular CCA-*l*1 (or CCA-elastic net) model by setting the group penalties to zeros.

In order to solve the model, an effective optimization algorithm, namely block cyclic coordinate descent algorithm, was designed. We built the model based on regularized SVD, which is similar to that used in sparse PCA [30], PMD (penalized matrix decomposition) [16] and 2-D penalized CCA [31]. Since the objective function used in the model was not convex, we re-formulated the model using the Lagrange form and transformed it into a biconvex optimization problem with the method in [16]. Unlike the existing sCCA models, the non-differentiability of sparse group lasso penalty used in the model poses a challenge for optimization and the standard coordinate descent algorithm will not work well. To this end, we designed an effective optimization method based on the block cyclic coordinate descent algorithm [24,26]. Our proposed group sparse CCA model can include both CCA-*l*1 and CCA-elastic net as special examples and hence the optimization method can be applied to solve these models as well.

The rest of the paper is organized as follows. The model and algorithm for our proposed group sparse CCA are described in section method. The performances of our model and other sCCA models are compared via both simulated and real data in section of results. The advantages and limitations of the proposed model are summarized in conclusion.

## Materials

### Gliomas data

Human gliomas are a group of brain tumors lacking an optimal treatment. Genomic techniques such as SNPs and gene expression arrays provide complementary information, which can be used together to improve the diagnosis and treatment of these tumors. Therefore, identifying the relationship between SNPs and gene expression is significant. The sCCA can be used for solving this problem by identifying correlations between gene expressions and SNPs.

Real data was downloaded from GEO database (GSE6109 for SNP genotype data and GSE4290 for gene expression). It was collected from 144 human gliomas containing 24 astrocytomas,46 oligodendrogliomas and 74 glioblastomas [32]. They include 58960 SNP measurements (XbaI-restricted DNA, Genechip Human Mapping 50 K arrays) and 54675 gene expression measurements (HG-U133 Plus 2.0). SNPs with >20% missing data were deleted and further missing data were imputed. The gene expression data were normalized. We assigned SNPs that are within the region 1 kb upstream of the transcription start sites (TSS) and to the end of the transcribed to be associated with a gene. By the canonical pathways from the Molecular Signatures Database (MSigDB), we identified the pathway named Reactome Downstream Events in GPCR Signaling (DEIGS), containing the most genes (432 genes). This pathway was analyzed by the proposed sCCA method. There are 897 gene expression measurements and 1106 SNPs included in this pathway.

### NCI60 data

Two expression datasets obtained on 60 cell lines from the National Cancer Institute (NCI) were used; see [33] for more details of data. A total 60 cell-lines were assayed for gene expression (the Staunton data set) and cDNA (the Ross data set). Those 60 cell-lines were derived from patients with leukemia (LE), melanomas (ME), cancers of ovarian (OV), breast (BR), prostate (PR), lung (LU), renal (RE), colon (CO) and central nervous system (CNS). The gene expression data were obtained using Affymetrix HG-U133A chip and cDNA data were profiled by spotted cDNA microarrays. Both data sets were normalized and pre-processed as described below.

In the Ross data set, 9703 spots were detected and those with more than 15% missing values were removed. The remaining missing values were imputed by a k-nearest neighbour approach [34]. The missing data was imputed by 16 nearest neighborhoods where the similarity between the two points was measured by Euclidean distance. The expression of these 16 neighborhoods would be weight averaged. The contribution of each neighborhood point was weighted by its similarity to the missing point. A subset of 1375 spots was further selected by filters used in [33]. In the Staunton data set, 7129 probe sets were used to screen each sample and 1517 probe sets having at least 500 average difference units across all cell lines were selected after a series of pre-processing steps described in [34].

To study the group effects of these biomarkers, we need to separate those genetic markers into different groups. We used hierarchical clustering method to cluster gene expression probes and cDNA spots based on the absolute values of their Pearson correlation coefficients and then grouped them with the threshold 0.2. In gliomas data set, 897 expression probes were grouped into 122 groups. SNPs were grouped by their gene annotations. Those SNPs located in the same gene region will be grouped together, resulting in 176 genes. In NCI60 data, there were 127 groups of expression probes and 139 groups of cDNA spots to be analyzed.

## Results

### Simulation

To investigate whether the group sparse CCA can improve the detection power when the group effect exists, we performed four simulated studies. We also used the simulation to compare its performance with the other two popular used sCCA methods (CCA-group and CCA-*l*1) under several conditions such as different sample size, varying number of correlated variables in the group and different correlations between two data sets.

In each study, two data sets ***X*** and ***Y***, consisting of *p* and *q* variables/features respectively were simulated. Data sets ***X*** and ***Y*** were divided into *G*_*X*_ and *G*_*Y*_ groups respectively. For the sake of simplicity, the sparsity of each group was set the same in both ***X*** and ***Y***. The sample size is *n*. To correlate the subset of variables in ***X*** with the subset of variables in ***Y***, we first set a latent variable ϒ = {*γ*_*i*_|*i* = 1, …, *n*} with distribution $N\left(0,\sigma_{\gamma }^{2}\right)$ to have the similar effect on the correlated variables in two data sets. Then ***X*** data set was generated by $x_{i}∼N\left(\theta_{X}\cdot \gamma_{i},\sigma_{e}^{2}\Sigma_{p\times p}\right)$ while ***Y*** data set by $y_{i}∼N\left(\theta_{Y}\cdot \gamma_{i},\sigma_{e}^{2}\Sigma_{q\times q}\right)$, where the vector *x*_*i*_ ∈ *R*^*p*^, *y*_*i*_ ∈ *R*^*q*^ are the observations of the *i*-th sample in ***X*** and ***Y***; $\theta_{X}=\left[\theta_{X}^{1},\theta_{X}^{2},\ldots ,\theta_{X}^{p}\right]$, $\theta_{Y}=\left[\theta_{Y}^{1},\theta_{Y}^{2},\ldots ,\theta_{Y}^{q}\right]$, $\theta_{X}^{j}\neq 0,\theta_{Y}^{k}\neq 0$, if *x*_*j*_, *y*_*k*_ are the correlated variables. Otherwise, the variables would be considered as random noise with zero means ($\theta_{X}^{j}=0$, $\theta_{Y}^{k}=0$); γ_*i*_ is the *i*-th observation of γ, and $\sigma_{\gamma }^{2}$ and $\sigma_{e}^{2}$ are the variances of γ and noise variable, and ∑ _*pxp*_ and ∑ _*qxq*_ are the variance-covariance matrices of each data set. We simulated the group effect within each dataset by referring to [20,21,35,36]. For each data set, we set the auto-regressive correlation between associated variables *i* and *j* within the same group to *ρ*^|*i* − *j*|^ and the correlation between different groups from uniform distribution Unif(0.2,0.4). *ρ* was preferred to be 0.5 according to [20]. The irrelevant variables were drawn from a normal distribution with mean zero and covariance from Unif(0,0.2).

If one data set (assuming ***Y***) contains categorical variables (*e*.*g*., SNP), we first simulated two set of continuous data ***X*** and ***Y***. Then the variables in ***Y*** were converted into categorical variables with three levels (*e*.*g*., -1, 0, 1) [37]. The minor allele frequency(MAF) *p* from the uniform distribution Unif(0.2,0.4) was selected randomly for each SNP; then based on Hardy-Weinberg Equilibrium(HWE), SNPs were converted according to the homozygous frequency *p*^2^, heterozygous frequency 2*p*(1-*p*), and homozygous frequency (1-*p*)^2^. For the irrelevant SNPs, they were sampled from the HapMap CEU panel (phase III) at chromosome 22 with 15329 SNPs(HWE < 0.001,MAF > =0.05) by the software HAPGEN2 [38].

We used the total true positive rate (TTPR), total false positive rate (TFPR) and total discordance (TD) to evaluate the performance of the modes. TTPR reflects the number of correctly identified correlated variables while TD is the number of incorrectly identified variables in both X and Y data sets. They are defined as

$\mathrm{TTPR}=\frac{\mathrm{TP}\_X+\mathrm{TP}\_Y}{\mathrm{TP}\_X+\mathrm{FN}\_X+\mathrm{TP}\_Y+\mathrm{FN}\_Y}$, $\mathrm{TFPR}=\frac{\mathrm{FP}\_X+\mathrm{FP}\_Y}{\mathrm{FP}\_X+\mathrm{TN}\_X+\mathrm{FP}\_Y+\mathrm{TN}\_Y}$

 and *TD* = *FP* _ *X* + *FN* _ *X* + *FP* _ *Y* + *FN* _ *Y*

where *TP*_*X* , *TP*_*Y*, *FP*_*X*, *FP*_*Y*, *FN*_*X*, *FN*_*Y* are true positives, false positives and false negatives in ***X*** and ***Y*** respectively. In each study, 50 replications were simulated and 5-fold cross validation was used for parameter selection in each replication.

The receiver operating characteristics (ROC) curve was also adopted for the comparison of three methods in identifying the correlated variables. The curve was drawn with TFPR versus TTPR and by varying the tuning parameters. For CCA-*l*1 and CCA-group methods, there are two parameters searched by a 10 × 10 grid λ takes the values: 0.04, 0.08, …, 0.4) with totally 100 parameter combinations. For group sparse CCA method, there are four parameters searched by a 10 × 10 × 10 × 10 grid of totally 10000 parameter combinations. Since group lasso penalty and *l*-1 norm penalty can both shrink the coefficient vectors, we adopted two steps to draw the ROC curve by varying sparsity level: first we obtain the optimal parameters based on the cross validation; then we vary one parameter while fixing the other parameters as the optimal ones to draw the curve. The average values over 50 replications were used to plot the ROC curves. The ROC curves of varying the first parameter were provided in Additional file 1.

Based on above descriptions, four simulation studies were conducted to investigate the performance of the methods in terms of recovering group structure (Simulation 1), the effect of group size (Simulation 2), the sample size (Simulation 3) and the true correlation value (Simulation 4).

### Simulation 1 the recovering accuracy of correlated variables using different CCA models

**Figure 1:**
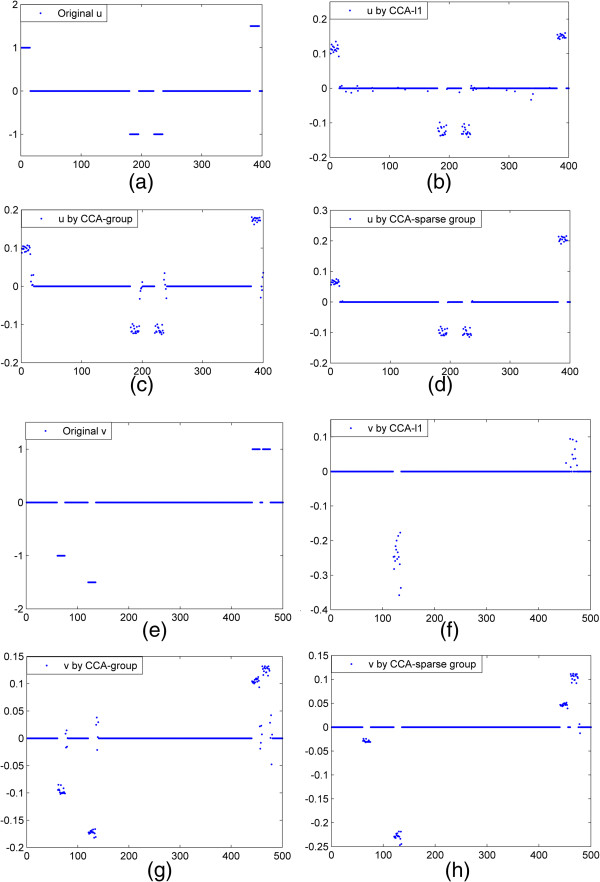
**A comparison of the performance of three sCCA methods. (a)** True ***u***; **(b-d)**. ***u*** recovered by CCA-*l*1, CCA-group and CCA-sparse group respectively. **(e) **True ***v***; **(f-h)**. ***v*** recovered by three SCCA methods.

We simulated *p* = 400 variables in data set ***X*** and *q* = 500 variables in data set ***Y***, which contain 60 correlated variables (defined as true variables) in each data set with the rest as noise. Data set ***X*** is divided into *G*_*X*_ = 20 groups and data set ***Y*** contains *G*_*Y*_ = 25 groups with the group size 20. The sample size is 100, σ_*γ*_ = 1 and*σ*_*e*_ = 0.5. We set the vector ***θ***_*X*_ to have 15 1 s, 30 **-**1 s, 15 1.5 s and the remaining 0 s; vector ***θ***_*γ*_ with length *q* have 15 -1 s, 15 –1.5 s, 30 1 s and the rest are 0 s. Each 15 non-zero coefficients are assigned randomly into one group along with the remaining 5 noise variables as shown in Figure 1(a, e). The correlated variables in each data set are assigned into four groups and correlated within each group. Figure 1(b-d, f-h) shows the results of recovered loading vectors ***u*** and ***v*** by CCA-*l*1, CCA-group and CCA-sparse group methods respectively. It can be seen that the CCA-sparse group method can better estimate true ***u*** and ***v*** than the other two methods. The CCA-*l*1selects more noise variables than true ***u***, increasing the false positive in Figure 1(b). Also, it misses out some true variables in Figure 1(f) when selecting ***v***. The CCA-group can better recover all the groups with true variables but also give more false positives in the groups.

### Simulation 2 the effect of the number of correlated variables in the group

**Figure 2:**
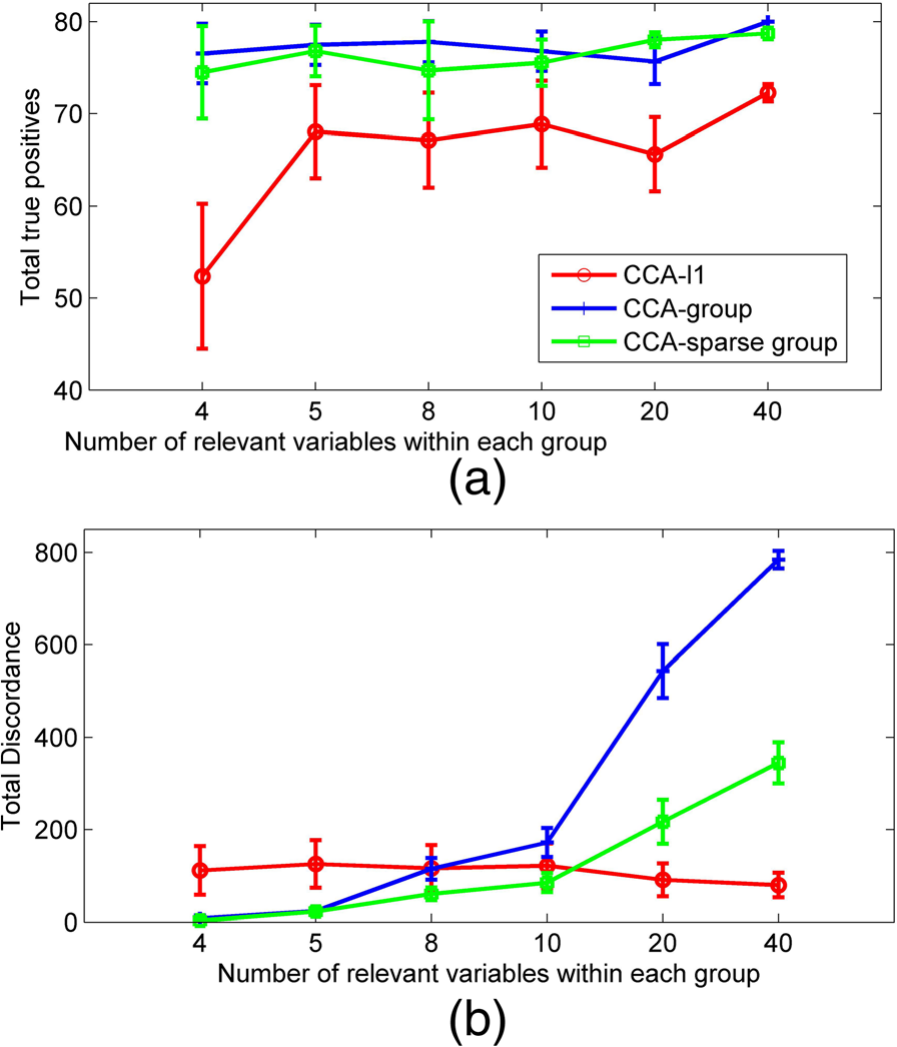
**The comparison of three methods for different group size. (a)** The total true positive recovered by CCA-*l*1,CCA-group and CCA-sparse group when group size changes by (4, 5, 8, 10,20, 40); **(b)** The total discordance of three methods when changing group size.

Variables can be distributed randomly in the data. Some of them could be grouped together while others are sparsely located in groups. We run this simulation to study the performance of the methods when the number of correlated variables within the group is changed. We simulated data set ***X*** with *p* = 400 variables and data set ***Y*** with *q* = 500 variables. 40 variables in each data set are correlated and the rest are noise variables. Data set ***X*** contains *G*_*X*_ = 40 groups and data set ***Y*** contains *G*_*Y*_ = 50 groups with the same group size of 10. The sample size is 100 and the standard deviation *σ*_γ_ = 1, *σ*_*e*_ = 0.3. In each data set, the number of groups containing correlated variables is set to be (4, 5, 8, 10, 20, 40) each time and each group contains (10, 8, 5, 4, 2, 1) true variables correspondingly. The averaged results over 50 replications are shown in Figure 2. When correlated variables are distributed in 4 groups, both CCA-group and CCA-group sparse models give much higher TTP while lower TD than those of CCA-*l*1. When the number of groups increases (true variables are more sparsely distributed into different groups), the TTP of CCA-group is still quite high but TD increases rapidly. The TTP of CCA-*l*1 will increase while having TD at a low level. The CCA-sparse group performs better and is more stable than other methods for having higher TTP and the lowest TD. When more correlated variables are assigned to the same group (*e*.*g*., 4 v.s 1 out of 10 variables), the CCA-sparse group model will obtain higher TTP and lower TD. This indicates that, if more true variables are grouped, the power of CCA-sparse group method will be increased and the false positives will be decreased (see Additional file 1).

### Simulation 3 the effect of sample size

**Figure 3:**
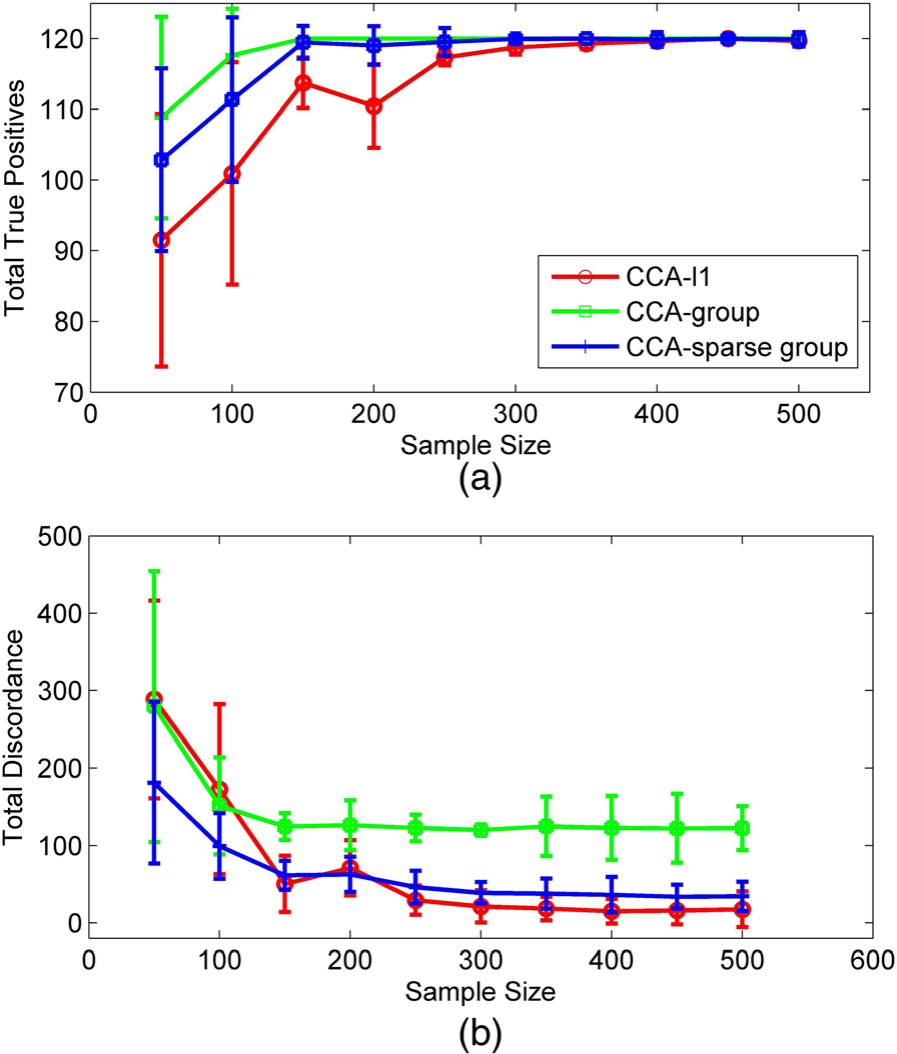
**A comparison of three methods for different sample size. (a) **The TTP (spell it out) value of using the CCA-*l*1,CCA-group and CCA-sparse group with sample size from 50 to 500; **(b)** The TD by the three methods with sample size from 50 to 500.

In this study, we discuss the effect of sample size on the recovery performance. We simulated data set ***X*** with *p* = 400 variables, *G*_***X***_ = 40 groups and data set ***Y*** with *q* = 500 variables, *G*_***Y***_ = 50 groups. The group size was fixed to be 10. 60 variables in each data set are correlated, which are distributed evenly into 6 groups. We then increase the sample size in step of 50 from *n* = 50 to 500 in order to compare the performance (*e*.*g*., discordance) of using different methods. Figure 3(a) shows the TTP with respect to different sample size. It can be seen that when the sample size increases, the TTP of all methods increases while the TD decreases. The CCA-group can achieve a better identification of true correlated variables than those using other two methods, but it also has more TD. The TTP of CCA-sparse group is less than that of CCA-group but more than that of CCA-*l*1. However, the TD by CCA-sparse group is much less than that of CCA-group. Especially when the sample size is large (>300), the TD of CCA-sparse group and CCA-*l*1 will stay at the low level. In addition, the TTP and TD of CCA-group and CCA-sparse group tend to have little changes when more than 150 samples are used, while those of CCA-*l*1 keep changing until almost 300 samples are used. The ROC curves (Additional file 1) at different sample sizes (50,200,300) well demonstrate that the CCA-sparse group can obtain a competitive power and sensitivity with much less samples than the other methods.

### Simulation 4 the performance of the methods influenced by noise

**Figure 4:**
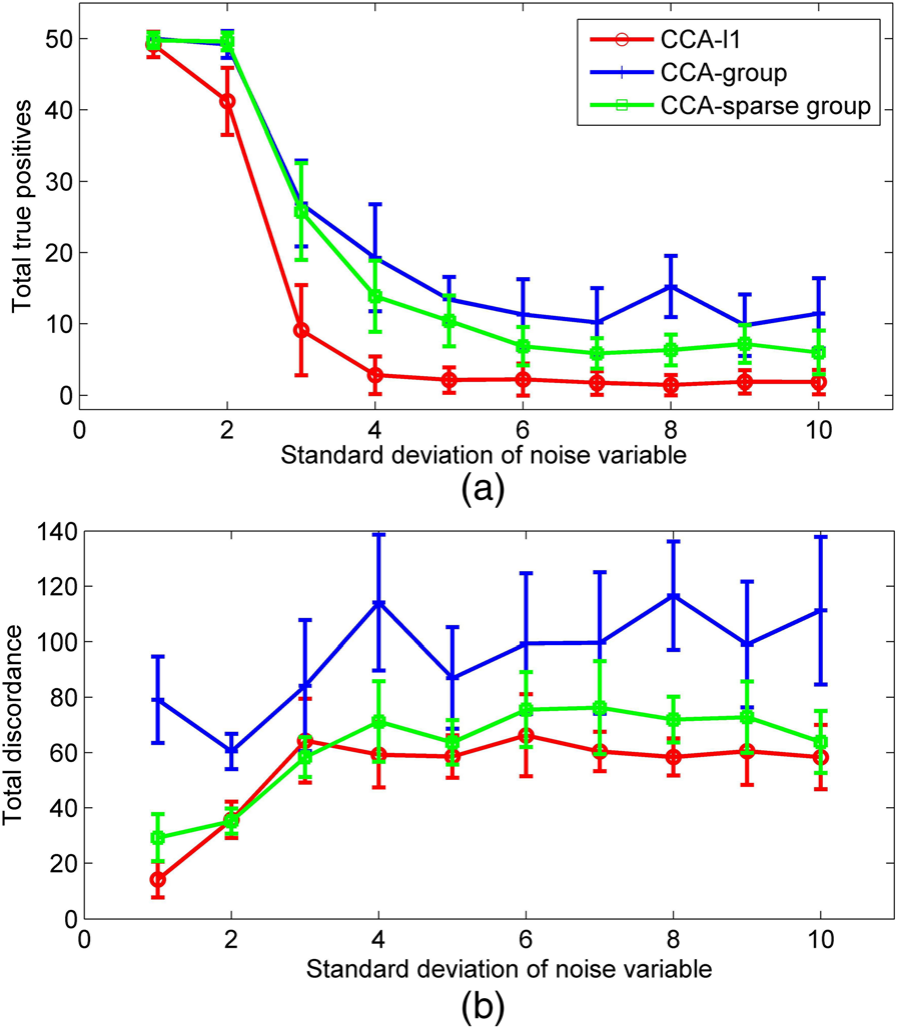
**A comparison of three methods for different correlation level influenced by noise. (a)** The value of total true positive obtained by three methods when the standard deviation of noise increases from 0.1 to 1, showing the highest correlation of true variables between two data sets within the range from 0.958 to 0.18. **(b)** The total discordance by three methods when the standard deviation of noise changes from 0.1 to 1.

Finally, we discuss the performance of three models under different noise levels, and study how noise affects the recovery of correlations between two data sets. We simulated data set ***X*** with *p* = 200 variables, *G*_***X***_ = 20 groups, and data set ***Y*** with *q* = 250 variables, *G*_***Y***_ = 25 groups, having the same group size 10. The correlation of different values is calculated between 20 variables in ***X*** and 30 variables in ***Y***. The sample size is 100 and *σ*_*γ*_ = 1. According to the estimation of the highest correlation in [7], there are several factors involved with estimating the highest correlation between the two data sets, *i.e.σ*_*γ*_, *σ*_*e*_, $\sum_{i}\alpha_{i}$, $\sum_{i}\beta_{i}$, the number of true variables in each data set, and the correlation among true variables within the same group. We fixed other factors but changed the standard deviation *σ*_*e*_ in the noise model from 0.1 to 1 with interval 0.1 to manipulate the correlation coefficient between two data sets. The highest correlation estimated (refer to [7]) is decreased from 0.958 to 0.18 accordingly From the results in Figure 4, we can see that when the true correlation increases due to the decrease of noise, more true variables can be recovered with less total discordance by all three methods. The CCA-group model can recover the most correlated variables but also has the highest total discordance. Compared to other two models, the CCA-*l*1 model has a lower TD but also the lowest TTP. The CCA-sparse group model can achieve comparable of TTP as that of the CCA-group while can have significantly reduced TD. Especially when *σ*_*e*_ < 0.4, the TD of CCA-sparse group decreases rapidly. The same result can be shown in ROC curves(*σ*_*e*_ = 0.2, 0.4, 0.8, see Additional file 1); CCA-sparse group is expected to give increasingly more power than CCA-*l*1 as well as better sensitivity than CCA-group when *σ*_*e*_ is reduced.

To test the computational complexity, we applied the methods on a simulated large data set with 1000 samples by 1000 gene expressions and 10000 SNPs. In one replicate, each single run time of three methods was on average 0.1444 sec, 0.1782 sec, and 0.1636 sec respectively. The time of running 5-fold cross validation based on the lambdas searching grid was 22.47 sec, 57.32 sec and 203.66 sec respectively. The memory usage was about 547.2 Mega bytes for all of them. The longer time of CCA-sparse group method was because of more parameters introduced in the model (*e*.*g*. two more parameters for controlling the sparsity within the groups, compared to CCA-group and CCA-*l*1 methods). The experiments were carried out on a desktop computer with a dual-core 2.8 GHz x86 64 bit processor, 6 GB memory. This shows that three sCCA methods are generally scalable and computationally affordable for large data sets.

### Application to real data analysis

### Comparison of three sCCA model sets

We applied the CCA-*l*1, CCA-group and CCA-sparse group to the analysis of these data sets for the purpose of comparisons. The comparisons were based on the correlations derived from the independent data sets, and the feature/variable selection in terms of feature overlapping by the methods, as well as the feature difference across the dimensions. Here each dimension indicates each pair of canonical variates. Each input data sets was normalized to have zero means in the column and unit variance to correct the scale differences from different datasets.

#### Independent correlation test

**Figure 5:**
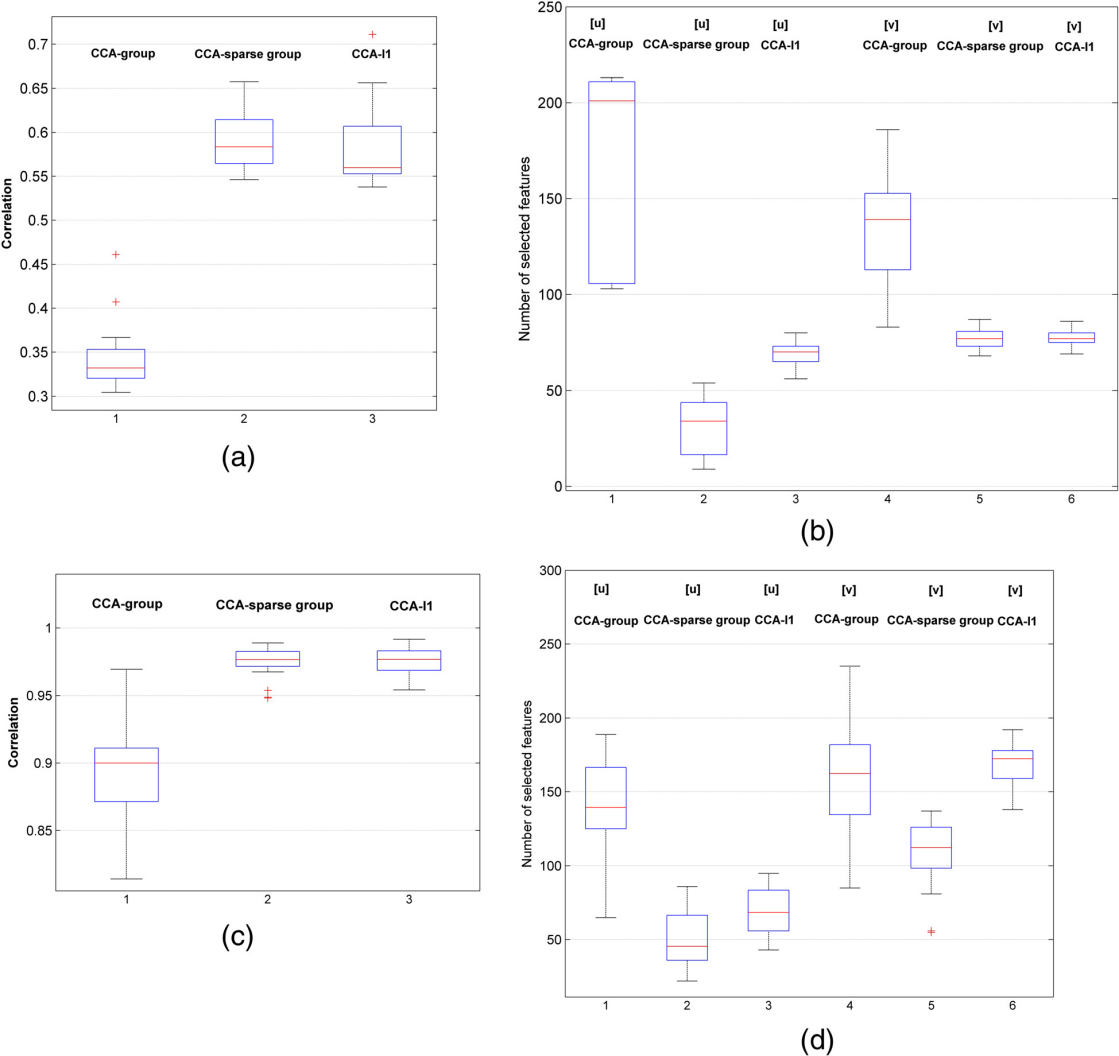
**The correlations and the features obtained by three sCCA methods based on 30 permutations of subjects. (a)**. The comparison of estimated correlations in glioma data; **(b)**. The comparison of number of selected feature in glioma data; **(c)**. The correlation comparison in NCI60 data; and **(d)** the feature selection comparison in NCI60 data (follow the same as **(a)** and **(b)**).

To test the performance of exploring the maximum correlation between two data sets using different methods, we divided the subjects into two groups: the training group and independent testing group. The training group was used to obtain the optimal parameters for the models while the testing group was used for testing the correlation between the two independent data sets. In the gliomas data, 100 subjects from 144 subjects were used for training and the rest 44 were used for testing. In NCI60 data set, 60 subjects were divided into 40 training subjects and 20 testing subjects. To alleviate the effect of subject difference, we permuted the subjects 30 times. The box-plot of the results is shown in Figure 5. The median correlation ± standard deviation obtained in gliomas data (Figure 5(a)) and NCI60 data (Figure 5(c)) by the CCA-sparse group (0.5829 ± 0.0392 in the gliomas data and 0.9765 ± 0.0075 in NCI60), CCA-*l*1(0.5527 ± 0.0454 and 0.9767 ± 0.0079) and CCA-group(0.3495 ± 0.0464 and 0.8999 ± 0.0297) are displayed respectively. The median correlations by the CCA-sparse group and CCA-*l*1 are higher than those of CCA-group in both gliomas and NCI60 data. In addition, the standard deviations of the correlations by the CCA-group model in both data sets are larger than those of the other two methods, which tends to select the whole group of features including some unexpected ones as shown in Figure 5(b, d). In the gliomas data (Figure 5(b)), the CCA-sparse group selects less number of features and obtain a larger correlation with the smallest standard deviation. The CCA-group finds the largest amount of features but obtains the smallest estimation of correlations. The CCA-*l*1 can get the less number of features with better estimate of the correlation, but is still a little less than that of CCA-sparse group. In the NCI60 data (Figure 5(d)), the CCA-*l*1 identified the similar correlation value to that of CCA-sparse group but had a higher standard deviation and selected more features. All of these figures indicate that the detection of correlations and features by the CCA-sparse group method has the lowest sensitivity to the samples. Compared to other methods, the CCA-sparse group can extract less number of features to achieve a higher correlation.

**Figure 6:**
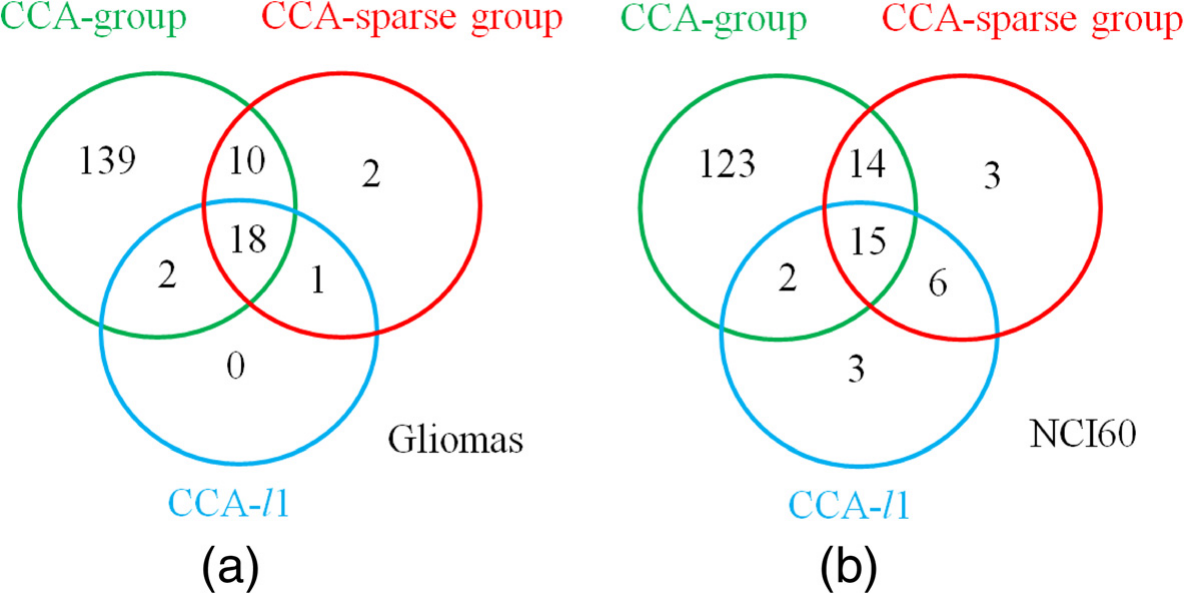
A comparison of the number of selected features with high frequency 20 out of 30 from the first canonical variate by three sCCA methods in the gliomas data (a) and the NCI60 data (b).

To further discuss the stability of the selected features during the permutation, we counted the number of features selected in 30 permutations by three methods and picked the features with the frequency of appearance greater than 20. Figure 6 shows the Venn diagrams of the selected features in ***u*** by three methods. It can be seen that, in both data sets, the features selected by CCA-*l*1 are almost included in those by other two methods, and overlap mostly with CCA-sparse group method. In addition, there are overlapped features (10 and 14 features) selected between the CCA-sparse group and CCA-group methods. Compared to CCA-*l*1, CCA-sparse group selects less features in ***u*** as shown in (Figure 5(b,d)); however, more features are selected with high frequency, which indicates higher stability of the method.

### Interpretation of the canonical variate pairs

Based on the independent test results, we use those optimal parameters as candidates for the cross validation to perform the correlation analysis on the complete subjects (including both training and testing samples) and analyze the biological significance of the canonical variates.

#### First and other canonical variate pairs

Table 1 shows three pairs of canonical variates for each data set by three sCCA methods. In both data sets, the correlations by the CCA-group are smaller than those of CCA-sparse group and CCA-*l*1. This agrees with the results in the independent analysis. In the gliomas data, the maximum correlation was, surprisingly obtained on the second dimension for all the methods. In the NCI60 data, all the correlations are very high in three dimensions, especially greater than 0.93 by the CCA-sparse group and CCA-*l*1 methods, which is consistent with the results in [2]. We can see that except the third canonical variate in NCI60 data, the CCA-sparse group can mostly identify the highest correlation with the least number of features, which confirms the analysis results in Figure 5. The maximum correlation is obtained on the first dimension for CCA-sparse group and the second pair for CCA-group and CCA-l1 methods. This trend of non-decreasing correlations in both data sets is not expected in the conventional CCA model, since it aims to extract the canonical variate with the maximum correlation at each step. This point was also discussed in [2] due to the optimization criterion and regularization term used in sCCA models. It can be found that the permutation of the correlation is in accord with the permutation of the dimensions.

**Table 1:** Correlations and the number of features from three pairs of loading vectors

**Data set**	**Model**	**dim1**			**dim2**			**dim3**			***p*(dim1)**
		**corr.**	**No.(u)**	**No.(v)**	**corr.**	**No.**	**No.**	**corr.**	**No.**	**No.**	
Glioma	CCA-group	0.3036	103	113	**0**.**3675**	153	93	0.3501	110	104	0.0005
	CCA-*l*1	0.5362	72	78	**0**.**6884**	83	76	0.5743	78	82	<0.0001
	CCA-sparse group	0.5903	53	47	**0**.**7023**	41	61	0.5728	67	60	<0.0001
NCI60	CCA-group	0.8425	189	184	**0**.**8943**	100	125	0.8310	125	115	<0.0001
	CCA-*l*1	**0**.**9645**	100	171	0.9527	67	120	0.9495	131	88	<0.0001
	CCA-sparse group	**0**.**9649**	45	71	0.9633	66	109	0.9355	97	108	<0.0001

To test the significance of the correlation estimation, we kept the selected features unchanged while permuting the subjects 10000 times in one data set to approximate the null distribution of the correlation with these selected features as in [2]. The *p* value was measured by the proportion of the correlations larger than the original correlation. We gave the *p* value of the correlation estimation using the first pair of canonical variates in Table 1. Almost all of the methods can derive the correlation based on the dimension with very low *p* values, which demonstrates the significance of the sparse CCA methods and the selected optimal parameters.

#### Comparison of selected features

As described in the algorithm, when multiple pairs of canonical variate exist, we derive the next pair of loading vectors which are assumed to be orthogonal to those previously identified ones. Therefore, there is a small degree of overlap of the selected features between two loading vectors (*i*.*e*. ***u***_*j*_ and ***u***_*j*+*1*_ or ***v***_*j*_ and ***v***_*j*+*1*_), which indicates that those selected features at each loading vector may carry different level of uncorrelated information. This orthogonal property could be kept in the sparse CCA methods but is not often the case; see Table 2. We calculated three pairs of loading vectors for each method and compared the features, which are overlapping between any two pairs of loading vectors. There are few features overlapped between the CCA-sparse group and CCA-*l*1 in almost all dimensions. Only 0 to 7 features are overlapping in the gliomas data and 0 to 8 features are overlapping in NCI60 data. Especially, there are no features overlapping across the three dimensions of ***u*** for CCA-sparse group in both data sets. However, for CCA-group, there are a large number of overlapping features. Since the feature selection with CCA-group is based on the group level, the overlapping of the individual features cannot be controlled.

**Table 2:** Comparison of the number of the commonly selected features in the three pairs of loading vectors

**Dataset**	**Glioma**			**NCI60**		
**Model**	**CCA-group**	**CCA- *l *1**	**CCA-sparse group**	**CCA-group**	**CCA- *l *1**	**CCA- sparse group**
[***u***]dim1-2	34	4	0	0	0	0
[***u***]dim1-3	0	5	0	29	4	0
[***u***]dim2-3	65	0	0	0	4	0
[***u***]dim 1-2-3	0	0	0	0	0	0
[***v***]dim1-2	0	1	2	27	3	1
[***v***]dim1-3	77	2	0	54	6	8
[***v***]dim2-3	0	6	7	0	5	1
[***v***]dim 1-2-3	0	0	0	0	0	0

## Discussion

### Biological interpretation of NCI60 data analysis

We give some discussions on graphical representations to study the discriminating ability of the selected features. The biological interpretations are provided by pathway analysis. Le Gao *et al*. performed the hierarchical clustering analysis of Ross and Staunton data sets in [2]. A better clustering of the cell lines can be obtained based on their tissue of origin with Ross data set. The 60 cell lines in Ross data set were mainly divided into three groups by their correlations: 1) cell lines with epithelia characteristics (mainly LE and CO, plus BR_MCF7 and BR_T47D); 2) cell lines with mesenchymal characteristics(mainly RE, CNS,LU and OV); and 3) ME cell lines (plus BR_MDAMB435 and BR_MDAN).

**Figure 7:**
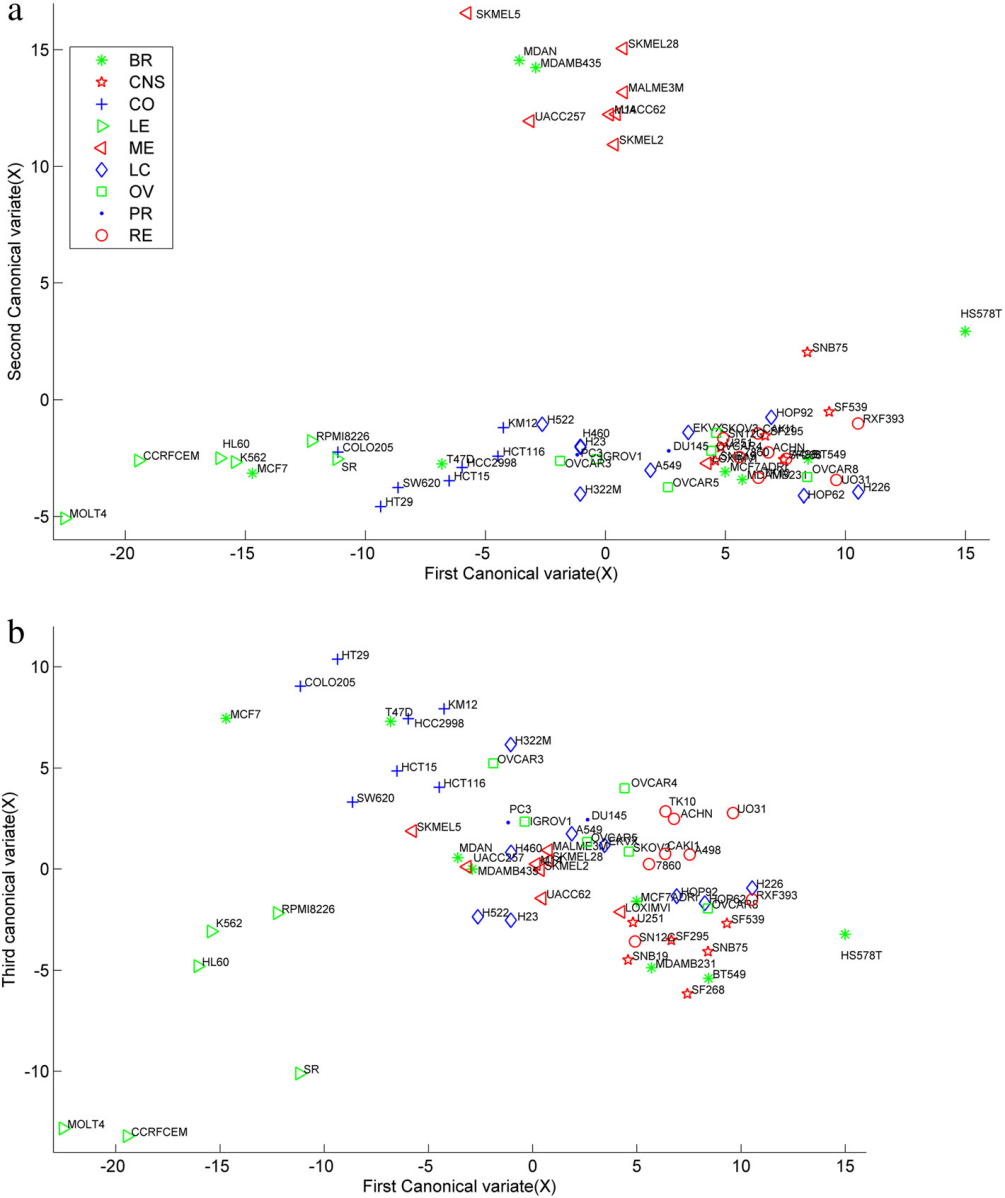
**NCI60 data: graphical representation of the samples with the canonical variate (*u*1,*u*2) (a) and (*u*1,*u*3) (b).** BR = Breast, CNS = Central Nervous System, CO = Colon, LE = Leukemia, ME = Melanoma, LU = Lung, OV = Ovarian, PR = Prostate, RE = Renal.

Based on these three groups of cell lines, we used graphical representation to evaluate whether the canonical variates and the corresponding identified features can represent different tumor cell lines. We computed three pairs of canonical variate by the CCA-sparse group method and drew the scatter plot of the samples using the first *v*.*s*. the second canonical variate (Figure 7(a)) and the first *v*.*s*. the third canonical variate (Figure 7(b)). Here each cell line was represented by three canonical variates. Each canonical variate was taken as one dimension of the cell line. It can be seen that the genes selected in the first dimension could help discriminate cell lines with epithelia from cells lines with mesenchymal characteristics; the features in the second dimension could discriminate themelanoma cell lines from the other cell lines and the features in the third dimension are opt to separate the CO cell lines from the LE cell lines. The similar representations can be derived for CCA-group and CCA-*l*1 method (not shown here). We found that there was a permutation for the dimension 1 and dimension 2 in the CCA-group method, which is consistent with the permutation of correlation in dimension 1 and 2 as shown in Table 2.

### Pathway analysis

**Figure 8:**
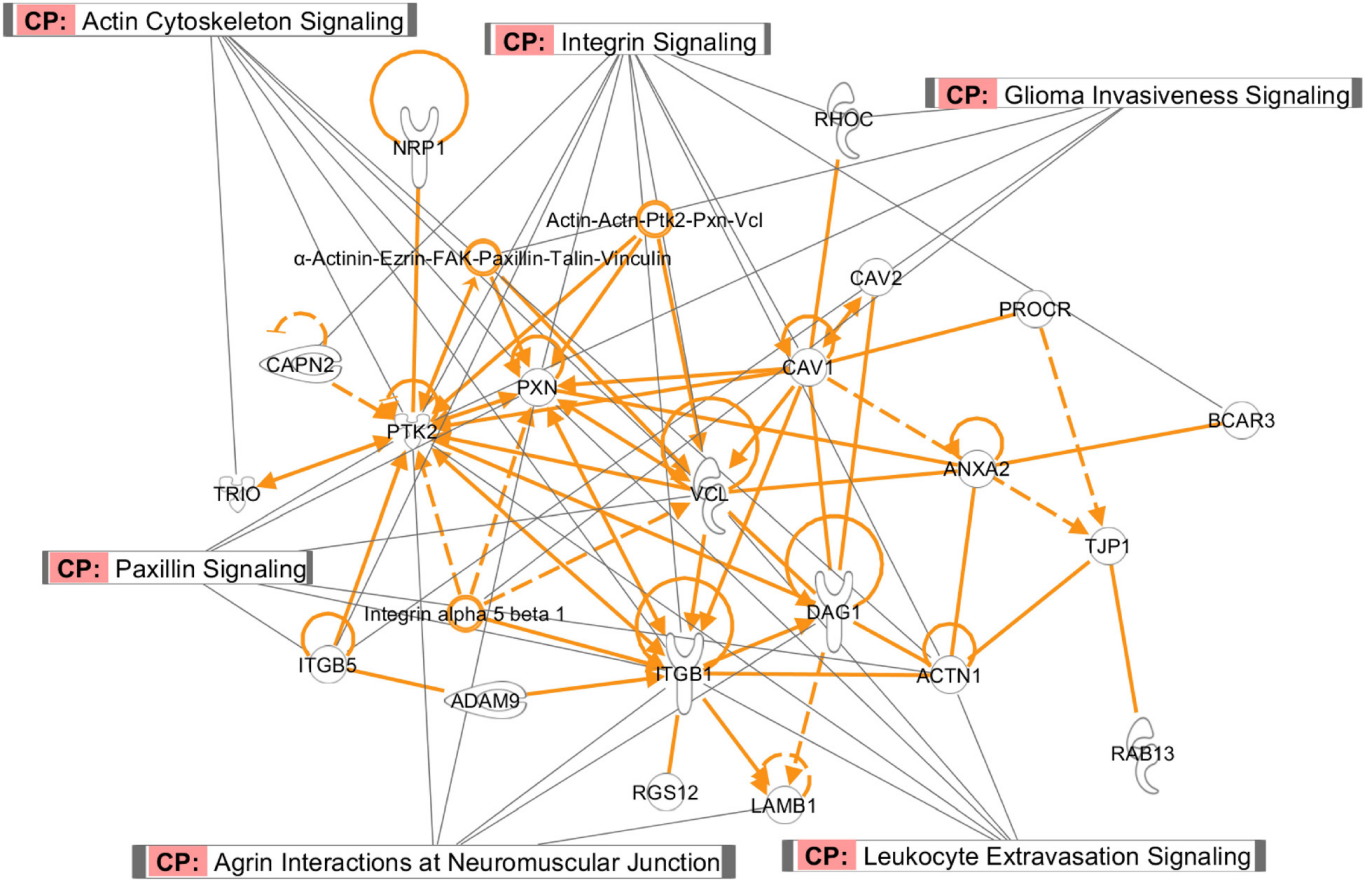
Pathway analysis of genes selected with the first dimension (variate) of CCA-sparse group model.

To further evaluate the biological significance of the features as well as the genes selected by the CCA-sparse group method, we used the Ingenuity Pathway Analysis (IPA: Ingenuity Pathways Analysis, http://www.ingenuity.com) to analyze those significant canonical pathways for discriminating different cell lines as shown in Figure 8. The genes in three dimensions by the CCA-sparse group were taken as input in IPA software to identify the corresponding pathways.

In the first dimension, the integrin signaling, paxillin signaling, agrin interactions at neuromuscular junction and actin cytoskeleton signaling pathways were identified. Those pathways play essential roles in some important biological processes including cellular movement, cell differentiation and cellular interactions with the cell-extracellular matrix [39-41]. Some genes, *i*.*e*. integrins and actinin and vinculin are over-expressed in RE and CNS cell lines, compared to LE and CO cell lines. The identified leukocyte extravasation signaling is known to be responsible for the leukocyte migration and might be related to the metastasis in the leukemia cell lines (LE) [42]. Several genes (i.e. ITGB5 and PTK2) in the glioma invasiveness signaling are over-expressed for the invasion and migration of glioma cells in the CNS cell lines [43]. In the second dimension, some genes including EGFR,MAPK9, MMP14 and CDKN2A from the Epithelial cell signaling in helicobacter pylori infection, p53 signaling, GnRH signaling and metabolic pathways are over-expressed in melanoma cells and involve with cellular growth, migration and metastasis [44-46]. In the third dimension, the tight junction signaling pathway (*i.e.* CLDN4, ACTN1,TJP1) was identified, which is consistent with epithelial characteristics of CO cell lines [47]. Leukocyte transendothelial migration pathway was also identified to separate LE cell lines from CO cell lines.

Although group sparse CCA can handle high number of variables with small sample size, the larger sample size can improve the results. From the simulation result, we can see the higher false positive and lower true positive when the sample size is too small.

In simulation, the group effect for relevant variables within the group is simulated with autoregressive correlation by *ρ*^|*i* − *j*|^. We chose the *ρ* value as 0.5 as suggested by [20]. The higher *ρ* value indicates higher correlation between variables correlation. The group effect for relevant variables between two groups is drawn from Unif(0.2,0.4). In group sparse CCA model, the between group correlation is expected to be low. If there is a strong correlation between two groups, similar to the lasso, it will result in the multi-collinearity issue. The correlation between two data sets may not be affected but the power of detecting correlated variables will decrease. In this case, we suggest to combine two highly correlated groups into one group or to incorporate the between group correlation information into the model.

In group sparse CCA model, the groups are assumed to be non-overlapping. This criterion can be met when grouping gene expressions by the hierarchical model or SNPs by their gene annotations. However, if we group gene set or SNPs by their pathway index, the group overlapping may exist. In this case, we can modify group sparse CCA by using the algorithm of Chen *et al.’s* or expand the overlapped genes (or SNPs) into both groups as discussed in [48].

## Conclusions

In this paper, we propose a group sparse CCA model to explore the correlation between two different types of genomic data. We solve the model with an efficient algorithm based on regularized SVD and block cyclic coordinate decent approach. The general model we propose can include CCA-*l*1, CCA-elastic net and CCA-group models as special examples. Our algorithm for solving CCA-*l*1 is very similar to that in [7] and we show that the algorithms for CCA-*l*1 and CCA-elastic net will converge to the same solution under a particular condition. We compare the performance of group sparse CCA method with CCA-*l*1 and CCA-group methods on simulation data under different conditions such as sample size, group size and the measurement of correlations. Undoubtedly, our CCA-sparse group method outperforms the existing ones by identifying features with more true positives while controlling the total discordance at a low level. The real data analysis on human gliomas and NCI60 data sets shows that the CCA-sparse group can explore significant correlations with smaller variance and the selected features shows higher stability. The graphical display and pathway analysis of NCI60 data indicates the selected features contain important information of different tumors. For these reasons, we believe that the CCA-sparse group method is an interesting and valuable approach for feature selection and correlation analysis.

## Methods

In this section, we first introduce sparse CCA model, based on which the group sparse model is presented. Then the numerical algorithms using the block cyclic coordinate descent to solve the model are described. Finally, the model and algorithm are extended to include the CCA-group model, CCA-*l*1 and CCA-elastic net models.

### Sparse CCA model

We denote the two sets of data with *n* samples by ***X*** and ***Y***, where ***X*** has *p* features and ***Y*** has *q* features, and usually *p*, *q*> > *n*. We assume that the columns of ***X*** and ***Y*** have been standardized to have mean zero and standard deviation one. The variance matrix of ***X*** and ***Y*** is denoted by ∑_XX_ and ∑_YY_ respectively, and the covariance between ***X*** and ***Y*** is denoted by ∑_XY_ or ∑_YX._ The CCA method aims to find two loading vectors or projections ***α***, ***β***, the linear combinations of variables in ***X*** and ***Y***, to maximize the correlation between ***α***^*t*^***X*** and ***β***^*t*^***Y*** as shown in (1):

(1) $$\underset{\alpha ,\beta }{\text{max}}\;\alpha^{t}\sum_{\mathrm{XY}}\beta \;s.t.\;\alpha^{t}\sum_{\mathrm{XX}}\alpha =1,\beta^{t}\sum_{\mathrm{YY}}\beta =1$$

The scales of ***α*** and ***β*** are set one since they have no effects on the correlation. This problem can be solved by the SVD of matrix ***K***

(2) $$K=\sum_{\mathrm{XX}}^{-1/2}\sum_{\mathrm{XY}}\sum_{\mathrm{YY}}^{-1/2}=\sum {}_{i}d_{i}u_{i}v_{i}$$

where *d*_*i*_ is the positive square root of the *i*-*th* eigen-value of ***K***^*t*^***K*** or ***KK***^*t*^ and sorted as $d_{1}^{2}\geq d_{2}^{2}\cdots \geq d_{r}^{2}\geq 0$ (*r* = *rank*(***X***^*t*^***Y***)). ***u***_*i*_, ***v***_*i*_ are the corresponding *i*-th eigenvectors. The loading vectors can be derived by

(3) $$\alpha_{i}=\sum_{\mathrm{XX}}^{-1/2}u_{i},\mathrm{and}\;\beta_{i}=\sum_{\mathrm{YY}}^{-1/2}v_{i}$$

the matrices $\sum_{\mathrm{XX}}^{-1/2}$ and $\sum_{\mathrm{YY}}^{-1/2}$ in (2) might be ill-conditioned because of the high dimensionality of data; conventional CCA is not working effectively for genomic data which have a large number of features but small number of samples. Previous work [7,17] has found that simply treating the covariance matrices as diagonal can do a better work. We adopt Witten *et al.*’s [17] method by replacing the covariance matrices with an identity matrix ***I*** and hence penalize the vectors ***u*, *v*** instead of the loading vectors ***α***, ***β***. By imposing sparse penalties on vectors ***u***, ***v***, we have the following sparse CCA optimization problem:

(4) $$\underset{u,v}{\text{min}}\;{∥K-duv^{t}∥}_{F}^{2}+\Psi \left(u\right)+\Phi \left(v\right)\;s.t.\;{∥u∥}_{2}^{2}=1,{∥v∥}_{2}^{2}=1$$

Where Ψ (***u***) and Ф (***v***) denote the penalized function on ***u*** and ***v*** respectively. Since (4) is not convex, by Witten *et al.*’s method, we relax it to the following optimization problem:

(5) $$\underset{u,v}{\text{min}}\;{∥K-duv^{t}∥}_{F}^{2}+\Psi \left(u\right)+\Phi \left(v\right)\;s.t.\;{∥u∥}_{2}^{2}\leq 1,{∥v∥}_{2}^{2}\leq 1$$

where ${∥u∥}_{2}^{2}=1,{∥v∥}_{2}^{2}=1$ should be satisfied when the solution is obtained (see optimization algorithms in Method).

### Group sparse CCA

It is natural to consider a group of features in concert to achieve higher power of detecting correlations in some applications including biomarker selection. Inspired by previous work described in introduction, we modify (5) to introduce the sparse group lasso penalty into the CCA model. For simplicity, we will only consider non-overlapping groups in this paper. Assume that features in ***X*** and ***Y*** are partitioned into *L* and *H* disjoint groups: $G_{X}^{1},G_{X}^{2},\ldots ,G_{X}^{L}$ and $G_{Y}^{1},G_{Y}^{2},\ldots ,G_{Y}^{H}$ respectively. The following group sparse CCA model is proposed to consider the group structure in the data:

(6) $$\begin{array}{l} \underset{u,v}{\text{min}}\;{∥K-duv^{t}∥}_{F}^{2}+\lambda_{1}\left(1-\tau_{1}\right){∥u∥}_{G}+\lambda_{1}\left(\tau_{1}\right){∥u∥}_{1}\\ +\lambda_{2}\left(1-\tau_{2}\right){∥v∥}_{G}+\lambda_{2}\left(\tau_{2}\right){∥v∥}_{1}s.t.\;{∥u∥}_{2}^{2}\leq 1,{∥v∥}_{2}^{2}\leq 1 \end{array}$$

where ${∥u∥}_{G}=\sum_{l=1}^{L}\omega_{l}{∥u_{l}∥}_{2},{∥v∥}_{G}=\sum_{h=1}^{H}\mu_{h}{∥v_{h}∥}_{2}$ are the group penalties to account for the joint effect of variables within the same group. ***u***_*l*_ and ***v***_*h*_ are the sub-vectors of ***u*** and ***v*** corresponding to the group *l* and *h* respectively. *τ*_1_ and *τ*_2_ are parameters to control the balance between group sparsity and individual feature sparsity. *λ*_1_ and *λ*_2_ are the tuning parameters to control the sparsity of the groups. *ω*_*l*_ and *μ*_*h*_ are the weights to adjust the group size difference. We set them to be *s*_*i*_^1/2^ , where *s*_*i*_ is the *i*-th group size.

The group penalty uses the non-diffentialbility of ∥***u***_*l*_∥_2_ at ***u***_*l*_ = ***0*** to set the coefficients of the group to be exact ***0*** and then the entire group of features will be dropped to achieve the group sparse. Similarly, for the *l*-1 norm penalty on the individual features, it also results in the sparsity of individual feature coefficients. Therefore, group sparse CCA method can select the features group-by-group as well as individual features within a group at the same time.

Because of these penalizations, the solution of group sparse CCA will use the following procedures: search for the first projection pair with a small subset of non-zeros groups of features through maximizing the correlation; then find the second sparse projection pair that maximizes the correlation but is irrelevant to the first pair. This process is not stopped until the *r*-th projection pair is gained.

In a particular case, when *τ*_1_ = *τ*_2_ = 0, (6) is reduced to the CCA-group method with only group lasso penalty as

(7) $$\Psi \left(u\right)=\lambda_{1}\sum_{l=1}^{L}\omega_{l}{∥u_{l}∥}_{2},\Phi \left(v\right)=\lambda_{2}\sum_{h=1}^{H}\mu_{h}{∥v_{h}∥}_{2}$$

As discussed above, without sparse penalty on individual features, CCA-group model can select features group by group and all features within a group will be retained.

### Optimization algorithms

The cyclic coordinate decent algorithm is efficient to solve generalized linear regression model [13,26,49], especially when handling large systems of equations. It finds one parameter at a time by fixing the other parameters. Similarly, a block cyclic coordinate decent algorithm is developed to estimate a block of parameters each time while fixing the other blocks of parameters. Taking the estimates of current step as the start of the next step in the algorithm, called ‘warm start’ makes the method to be remarkably efficient. Therefore, in this paper we use the block cyclic coordinate decent to solve the optimization problem in group sparse CCA model.

For simplicity, we decouple the problem in (5) into two simple biconvex optimizations: 1) when ***v*** is fixed, (5) is a classical convex optimization with respect to ***u***; 2) when ***u*** is fixed, it is the optimization with respect to ***v***. The initial value of ***u*** and ***v*** can be derived from the classical CCA decomposition. Therefore, the problem in (5) can be solved by the following algorithm:

1) Initialize ***u*** and ***v*** to have unit *l*-2 norm;

2) Solve ***u***, ***v*** using the following iterations until it converges:

a) Fix ***v*** = ***v***^*j* − 1^,

$$u^{j}\leftarrow {\arg\text{min}}_{u,d}{∥K-duv^{t}∥}_{F}^{2}+\Psi \left(u\right)\;s.t.\;{∥u∥}_{2}^{2}\leq 1$$

b) Fix ***u*** = ***u***^*j*^,

$$v^{j}\leftarrow {\arg\text{min}}_{v,d}{∥K-duv^{t}∥}_{F}^{2}+\Phi \left(v\right)\;s.t.\;{∥v∥}_{2}^{2}\leq 1$$

c) *d*^*j*^ ← *tr*(***Kv***^*j*^(***u***^*j*^)^*t*^) or *d*^*j*^ ← *tr*(***K***^*t*^***u***^*j*^(***v***^*j*^)^*t*^).

3) Update the remaining matrix ***K*** ← ***K*** − *d*^*j*^***u***^*j*^(***v***^*j*^)^*t*^; go to Step 1) to obtain the next pair of loading vectors (***u***^***j*+*1***^,***v ***^***j*+*1***^).

For Step a), the objective function can be decomposed as:

(8) $$\begin{array}{ll} \;{∥K-duv^{t}∥}_{F}^{2} & =\mathrm{tr}\left(\left(K-duv^{t}\right){\left(K-duv^{t}\right)}^{t}\right)\\ & =\mathrm{tr}\left(KK^{t}\right)-2\times d\times \mathrm{tr}\left(\mathrm{Kv}u^{t}\right)+d^{2} \end{array}$$

Similar decomposition can be done in Step b). Since the matrix ${KK}^{t}$ is known, *d* can be derived from *tr*(**Kv**^*j*^(***u***^*j*^)^*t*^) in a) or *tr*(***K***^*t*^***u***^*j*^(***v***^*j*^)^*t*^) in b). We translate the equality condition imposed on Step a) and b) into the Lagrange form by multiplying it with a Lagrange factor ∆ such that ${∥u∥}_{2}^{2}=1$ and ${∥v∥}_{2}^{2}=1$ is satisfied. Therefore, a) (similarly for b)) can be changed as:

(9) $$\begin{array}{ll} \text{Fix} & v=v^{j-1},\\ & u^{j}\leftarrow \underset{u}{\arg\text{min}}-\mathrm{tr}\left(\mathrm{Kv}u^{t}\right)+\Psi \left(u\right)+\Delta \left({∥u∥}_{2}^{2}-1\right) \end{array}$$

For the group sparse CCA model, the Lagrange form in (9) becomes:

(10) $$\begin{array}{l} \underset{u}{\text{min}}\;-\mathrm{tr}\left(\mathrm{Kv}u^{t}\right)+\lambda_{1}\left(1-\tau_{1}\right)\sum_{l=1}^{L}\omega_{l}{∥u_{l}∥}_{2}\\ \;+\lambda_{1}\left(\tau_{1}\right){∥u∥}_{1}+\Delta \left({∥u∥}_{2}^{2}-1\right) \end{array}$$

Where ∆ is the parameter to make ${∥u∥}_{2}^{2}=\sum_{l=1}^{L}u_{l}^{2}=1$.

A block cyclic coordinate decent algorithm is then used to solve (10). For group *k* = *1*,*2*,…,*L*, each group will be inspected first. If a group is selected, we will select each variable in the group by the coordinate decent algorithm with the soft-threshold. Since the optimization is convex, the optimal solution of (10) can be determined by the sub-gradient equation,

(11) $$\begin{array}{l} {\left(\mathrm{Kv}\right)}^{\left(k\right)}-2\Delta {\hat{u}}^{\left(k\right)}\;=\;\lambda_{1}\left(1-\tau_{1}\right)\omega_{k}\xi +\lambda_{1}\left(\tau_{1}\right)\Gamma ,\\ \Gamma_{j}=\left\{\begin{array}{l} \mathrm{sign}\left({\hat{u}}_{j}^{\left(k\right)}\right),\mathrm{if}\;{\hat{u}}_{j}^{\left(k\right)}\neq 0\\ \in \left\{\Gamma_{j}|\left|\Gamma_{j}\right|\leq 1\right\},\mathrm{if}\;{\hat{u}}_{j}^{\left(k\right)}=0 \end{array}\right),j=1,2,\ldots ,l_{k}\;\mathrm{and}\\ \xi =\left\{\begin{array}{l} \frac{{\hat{u}}^{\left(k\right)}}{{∥{\hat{u}}^{\left(k\right)}∥}_{2}},\mathrm{if}\;{\hat{u}}^{\left(k\right)}\neq 0\\ \in \left\{\xi |{∥\xi ∥}_{2}\leq 1\right\},\mathrm{if}\;{\hat{u}}^{\left(k\right)}=0 \end{array}\right) \end{array}$$

We can see that the coefficient vector of the *k*-th group ${\hat{u}}^{\left(k\right)}=0$ will be satisfied if

(12) $${∥S\left({\left(\mathrm{Kv}\right)}^{\left(k\right)},\lambda_{1}\tau_{1}\right)∥}_{2}\leq \lambda_{1}\left(1-\tau_{1}\right)\omega_{k}$$

Otherwise ${\hat{u}}^{\left(k\right)}$ can be updated by

(13) $${\hat{u}}^{\left(k\right)}\leftarrow \frac{Sg^{\left(k\right)}\left(\mathrm{Kv}\right)}{\Delta },\;Sg^{\left(k\right)}\left(\mathrm{Kv}\right)=\frac{1}{2}\left[S\left({\left(\mathrm{Kv}\right)}^{\left(k\right)},\lambda_{1}\tau_{1}\right)-\lambda_{1}\left(1-\tau_{1}\right)\omega_{k}\frac{S\left({\left(\mathrm{Kv}\right)}^{\left(k\right)},\lambda_{1}\tau_{1}\right)}{{∥S\left({\left(\mathrm{Kv}\right)}^{\left(k\right)},\lambda_{1}\tau_{1}\right)∥}_{2}}\right]$$

where *Δ* = ∥[*Sg*^1^(***Kv***), *Sg*^2^(***Kv***), …, *Sg*^*k*^(***Kv***)]∥_2_ to make ${∥\hat{u}∥}_{2}^{2}=1$, and S(·) is the soft-threshold function. A detailed description of the algorithm for solving group lasso CCA is shown in Additional file 2 A.

For the CCA-group model, i.e., a special case of group sparse CCA when *τ* = 0, the soft-threshold operator in (12) could be reduced to the following simple form:

(14) $${∥S\left({\left(\mathrm{Kv}\right)}^{\left(k\right)},\lambda_{1}\tau_{1}\right)∥}_{2}={∥{\left(\mathrm{Kv}\right)}^{\left(k\right)}∥}_{2}$$

Substituting (14) into (12), we can get the solution of CCA-group lasso model.

### Extension to the other models

From the general sparse CCA formula (5), we can derive the CCA-*l*1 and CCA-elastic net models as special examples.

The CCA-*l*1 is obtained when using the *l*-1 norm as the penalty,

(15) $$\Psi \left(u\right)=\lambda_{1}{∥u∥}_{1},\;\Phi \left(v\right)=\lambda_{2}{∥v∥}_{1}$$

The CCA-elastic net model is obtained by using the elastic net penalization,

(16) $$\begin{array}{l} \Psi \left(u\right)=\lambda_{1}\left(1-\tau_{1}\right){∥u∥}_{2}^{2}+\lambda_{1}\left(\tau_{1}\right){∥u∥}_{1},\\ \Phi \left(v\right)=\lambda_{2}\left(1-\tau_{2}\right){∥v∥}_{2}^{2}+\lambda_{2}\left(\tau_{2}\right){∥v∥}_{1} \end{array}$$

where *τ*_1_, *τ*_2_ are the parameters to control the trade-off between *l*-2 norm and *l*-1 norm penalized terms. The cyclic coordinate decent algorithm can also be applied to solve (15) and (16) and the following fact holds:

#### The equivalence of the solution

Let $\left(u_{l1}^{*},v_{l1}^{*}\right)$, $\left(u_{\mathrm{eNet}}^{*},v_{\mathrm{eNet}}^{*}\right)$ be the optimal loading vectors by the CCA with the *l*-1 norm and elastic net penalization respectively. When the regularization parameters satisfy: $\lambda_{\mathrm{eNet}}^{*}\tau_{1}=\lambda_{l1}^{*}$, with the same iteration procedure, the solutions for the loading vectors will be equal, *i*.*e*., $u_{l1}^{*}=u_{\mathrm{eNet}}^{*},v_{l1}^{*}=v_{\mathrm{eNet}}^{*}$.

The discussion of this relation is given in Additional file 2 B. Roughly speaking, if substituting (14) and (15) into (9), we will see that the condition on ***u*** with *l*-1 norm penalty has the similar estimate as that with the elastic net. In addition, under the *l*-1 norm penalization, (11) is very similar to the algorithm in Parkhomenko *et al.*[7]. Both of them use the soft-thresholding to obtain the sparse solution while the only difference is in the use of the tuning parameter. Our CCA-*l*1 is also similar to the PMD in [16], where the regularized SVD was used to solve sparse PCA and then extended to solve CCA.

### Tuning parameters selection

The group sparse CCA model uses parameters (*λ*_1_, *λ*_2_, *τ*_1_, *τ*_2_) and soft-thresholding in the algorithm to obtain the non-zero variables and the number of groups. For the sake of simplicity, we fix the sum of two penalties as *λ*_1_ and *λ*_2_ and use *τ*_1_, *τ*_2_ as a balance between group penalty and single variable penalty respectively. The *k*-fold cross-validation was recommended by Waaijenborg *et al*. [37] and Parkhomenko *et al*. [7] for parameter selection. We choose the parameters that can minimize the mean difference between the canonical correlations obtained with the training and testing subsets as (17).

(17) $$\Delta_{\mathrm{corr}}=\frac{1}{k}\sum_{i=1}^{k}\left|\mathrm{cor}\left(X_{-i}u^{-i},Y_{-i}v^{-i}\right)-\mathrm{cor}\left(X_{i}u^{-i},Y_{i}v^{-i}\right)\right|$$

This criterion determines the number of variables which tend to have the same correlations in both training and testing subsets.

## Competing interests

The authors declare that they have no competing interest.

## Authors’ contributions

DDL designed the model and developed the algorithm. DDL, JGZ and JYL performed the simulation and real data processing. VDC, HWD and YPW participated in the design of the study and writing of the manuscript. All authors read and approved the final manuscript.

## Supplementary Material

Additional file 1 Contains the results of ROC curves drawn by changing the penalty parameter values under different simulation studies. Click here for file

Additional file 2 **Contains the algorithm for solving group sparse CCA model and the proof of the equivalence of the solutions by CCA-*l*1 and CCA-elastic net methods.** Click here for file
