# Supplementary material for: Group sparse canonical correlation analysis for genomic data integration
Source: BMC Bioinformatics. 2013 Aug 12;14:245. doi: 10.1186/1471-2105-14-245 (PMC3751310; doi:10.1186/1471-2105-14-245)
Supplement: Additional file 1 — Contains the results of ROC curves drawn by changing the penalty parameter values under different simulation studies. [file 1471-2105-14-245-S1.pdf]

Additional file 2: The ROC curves for correlated variables detection by CCA-*l*1, CCA-group and CCA-sparse group methods.

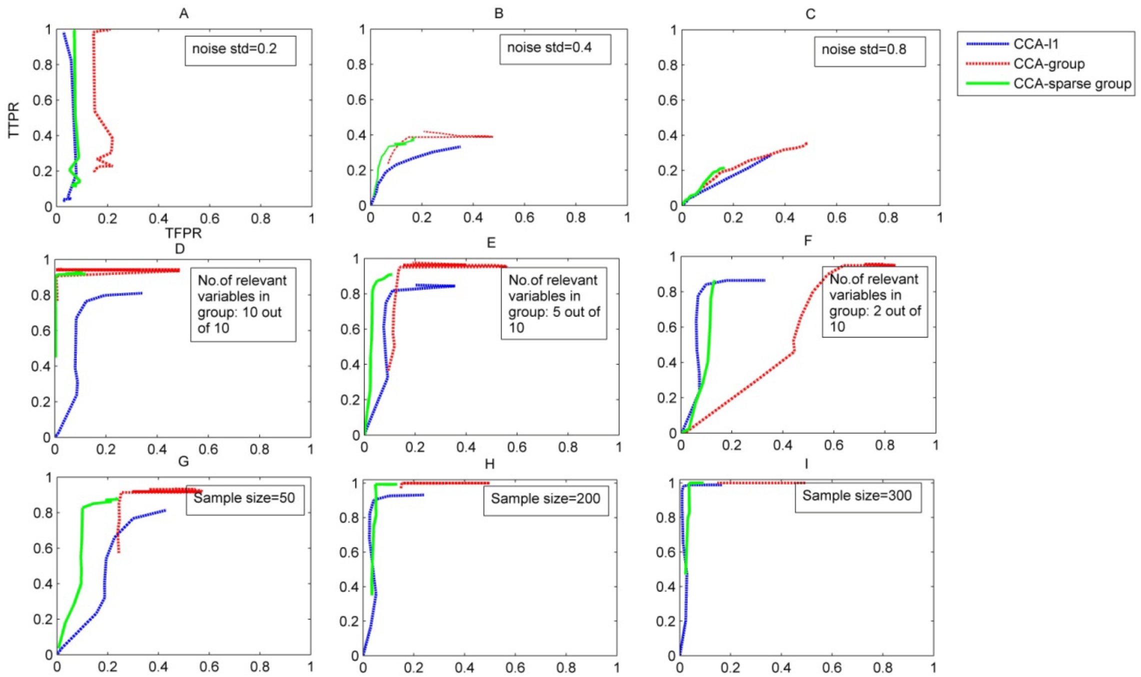

A-C: The comparison of three methods when simulating the noise variables with different standard deviation: noise std=0.2,0.4 and 0.8.

D-F: the comparison of methods by changing the number of correlated variables in the group.

G-I: the comparison of methods under different sample size(50,200,300).
