# Supplementary material for: Group sparse canonical correlation analysis for genomic data integration
Source: BMC Bioinformatics. 2013 Aug 12;14:245. doi: 10.1186/1471-2105-14-245 (PMC3751310; doi:10.1186/1471-2105-14-245)
Supplement: Additional file 2 — Contains the algorithm for solving group sparse CCA model and the proof of the equivalence of the solutions by CCA-l1 and CCA-elastic net methods. [file 1471-2105-14-245-S2.pdf]

Appendix:

A. Solving the CCA group lasso by the block coordinate decent algorithm:

The CCA with sparse group lasso penalization is a non-convex optimization problem described as follows:

$$\min_{\boldsymbol{u}} -tr\left(\boldsymbol{Kvu}^t\right)+\lambda_1\left(1-\tau_1\right)\sum_{l=1}^{L}\omega_l\left\|\boldsymbol{u}_l\right\|_2+\lambda_1\left(\tau_1\right)\left|\boldsymbol{u}\right|_1+\Delta\left(\left\|\boldsymbol{u}\right\|_2^2-1\right)\ (1)$$

where $\boldsymbol{u}=\left[\boldsymbol{u}^1,...\boldsymbol{u}^k...,\boldsymbol{u}^L\right]$, L is the number of groups in $\boldsymbol{X}$. By the coordinate decent, in each step, we will check the update within each group cyclically by fixing the coefficients of the other groups. We assume that $\hat{\boldsymbol{u}}$ is the estimate of the solution, for the group *k*; by the sub-gradient of (1), $\hat{\boldsymbol{u}}^{(k)}$ must satisfy:

$$\left(\boldsymbol{Kv}\right)^{(k)}-2\Delta\hat{\boldsymbol{u}}^{(k)}=\lambda_1\left(1-\tau_1\right)\omega_k\xi+\lambda_1\left(\tau_1\right)\Gamma$$

where $\xi=\begin{cases}\dfrac{\hat{\boldsymbol{u}}^{(k)}}{\left\|\hat{\boldsymbol{u}}^{(k)}\right\|_2}, if\ \hat{\boldsymbol{u}}^{(k)}\neq 0\\ \in\left\{\xi\mid\left\|\xi\right\|_2\leq 1\right\}, if\ \hat{\boldsymbol{u}}^{(k)}=0\end{cases}$, $\Gamma_j=\begin{cases}sign\left(\hat{\boldsymbol{u}}_j^{(k)}\right), if\ \hat{\boldsymbol{u}}_j^{(k)}\neq 0\\ \in\left\{\Gamma_j\mid\left|\Gamma_j\right|\leq 1\right\}, if\ \hat{\boldsymbol{u}}_j^{(k)}=0\end{cases}$, $j=1,...,l_k$ (2)

l_k is the number of variables within group *k*.

Substituting (2) into (1), we can see that $\hat{\boldsymbol{u}}^{(k)}=0$ will be satisfied if

$$\left\|S\left(\left(\boldsymbol{Kv}\right)^{(k)},\lambda_1\tau_1\right)\right\|_2\leq\lambda_1\left(1-\tau_1\right)\omega_k\quad (3)$$

Else $\boldsymbol{u}^{(k)}$ satisfies:

$$S\left(\left(\boldsymbol{Kv}\right)^{(k)},\lambda_1\tau_1\right)=\lambda_1\left(1-\tau_1\right)\omega_k\frac{\hat{\boldsymbol{u}}^{(k)}}{\left\|\hat{\boldsymbol{u}}^{(k)}\right\|_2}+2\Delta\hat{\boldsymbol{u}}^{(k)}\qquad (4)$$

where $S\left(y,\lambda\right)$ is the soft-thresholding operator given by below:

$$sign\left(y\right)\left(\left|y\right|-\lambda\right)_+=\begin{cases}y-\lambda\ \ if\ \ y>0\ and\ \left|y\right|>\lambda\\ y+\lambda\ \ if\ \ y<0\ and\ \left|y\right|>\lambda\ (5)\\ 0\qquad if\ \left|y\right|\leq\lambda\end{cases}$$

Taking the *l*-2 norm of both sides,

$$\left\|\boldsymbol{u}^{(k)}\right\|_2=\frac{1}{2\Delta}\left(\left\|S\left(\left(\boldsymbol{Kv}\right)^{(k)},\lambda_1\tau_1\right)\right\|_2-\lambda_1\left(1-\tau_1\right)\omega_k\right)(6)$$

So substituting (6)into (4), we can get the update of $\hat{\boldsymbol{u}}^{(k)}$ as follows:

$$\hat{\boldsymbol{u}}^{(k)}\leftarrow\frac{1}{2\Delta}\left[S\left(\left(\boldsymbol{Kv}\right)^{(k)},\lambda_1\tau_1\right)-\lambda_1\left(1-\tau_1\right)\omega_k\frac{S\left(\left(\boldsymbol{Kv}\right)^{(k)},\lambda_1\tau_1\right)}{\left\|S\left(\left(\boldsymbol{Kv}\right)^{(k)},\lambda_1\tau_1\right)\right\|_2}\right]\qquad (7)$$

Suppose $Sg^{(k)}\left(\boldsymbol{Kv}\right)=\frac{1}{2}\left[S\left(\left(\boldsymbol{Kv}\right)^{(k)},\lambda_1\tau_1\right)-\lambda_1\left(1-\tau_1\right)\omega_k\frac{S\left(\left(\boldsymbol{Kv}\right)^{(k)},\lambda_1\tau_1\right)}{\left\|S\left(\left(\boldsymbol{Kv}\right)^{(k)},\lambda_1\tau_1\right)\right\|_2}\right]$, then (7) can be rewritten as:

$$\hat{\boldsymbol{u}}^{(k)}\leftarrow\frac{Sg^{(k)}\left(\boldsymbol{Kv}\right)}{\Delta}\ (8)$$

After updating the whole groups in each step, we choose the parameter $\Delta=\left\|Sg^{(1)}\left(\boldsymbol{Kv}\right),Sg^{(2)}\left(\boldsymbol{Kv}\right),...,Sg^{(L)}\left(\boldsymbol{Kv}\right)\right\|$ to make $\left\|\hat{\boldsymbol{u}}\right\|_2^2=1$.

B. Proof of the equivalence of the solution

In CCA with the $l1$ norm penalization, we seek $\boldsymbol{u}$ by solving the following optimization problem:

$$\min_{\boldsymbol{u}} -tr\left(\boldsymbol{Kvu}^t\right)+\lambda_1\|\boldsymbol{u}\|_1+\Delta\left(\|\boldsymbol{u}\|_2^2-1\right) \quad (9)$$

Similarly, in the CCA-elastic net method, we seek $\boldsymbol{u}$ by minimizing the following object function:

$$\min_{\boldsymbol{u}} -tr\left(\boldsymbol{Kvu}^t\right)+\lambda_1\left(1-\tau_1\right)\|\boldsymbol{u}\|_2^2+\lambda_1\left(\tau_1\right)\|\boldsymbol{u}\|_1+\Delta\left(\|\boldsymbol{u}\|_2^2-1\right) \quad (10)$$

Suppose $F\left(\boldsymbol{u},\Delta\right)$ is the Lagrange function.The sub-gradient of function in (9) and (10) with respect to $\boldsymbol{u}$ is

$\nabla_{\boldsymbol{u}}F\left(\boldsymbol{u},\Delta\right)=2\Delta\boldsymbol{u}-\boldsymbol{Kv}+\Gamma_j$ (11) and

$\nabla_{\boldsymbol{u}}F\left(\boldsymbol{u},\Delta\right)=2\left(\Delta+\lambda_1\left(1-\tau_1\right)\right)\boldsymbol{u}-\boldsymbol{Kv}+\Gamma_j$ (12)

where $\Gamma_j=\begin{cases} sign\left(\boldsymbol{u}_j\right), if\ \boldsymbol{u}_j\neq 0 \\ \in\left\{\Gamma_j \mid \left|\Gamma_j\right|\leq 1\right\}, if\ \boldsymbol{u}_j=0 \end{cases}, j=1,...,p$.

By the coordinate decent, each $\hat{\boldsymbol{u}}_j$ from (11) and (12) will be estimated with cycles by the following update respectively:

$\hat{u}_j \leftarrow \frac{S\left(\left(\boldsymbol{Kv}\right)_j,\lambda_1\right)}{2\Delta}$ (13) and

$\hat{u}_j \leftarrow \frac{S\left(\left(\boldsymbol{Kv}\right)_j,\lambda_1\tau_1\right)}{2\left(\Delta+\lambda_1\left(1-\tau_1\right)\right)}$.(14)

Since the parameter Δ needs to be chosen such that $\|\boldsymbol{u}\|_2^2=1$ is satisfied, we modify (13) to get:

$\hat{u}_j \leftarrow \frac{S\left(\left(\boldsymbol{Kv}\right)_j,\lambda_1\right)}{\left\|S\left(\left(\boldsymbol{Kv}\right)_j,\lambda_1\right)\right\|_2}$ (15)

Similarly, (14) will be modified as follows:

$\hat{u}_j \leftarrow \frac{S\left(\left(\boldsymbol{Kv}\right)_j,\lambda_1\tau_1\right)}{\left\|S\left(\left(\boldsymbol{Kv}\right)_j,\lambda_1\tau_1\right)\right\|_2}$ (16)

It can be seen that (16) is similar to (15) with only the difference of tuning parameter. Under the convex optimization, both (15) and (16) will converge to the same optimal solution when the parameter of (16) is τ_1 times that of (15). Proof is completed.
